# Proteases and Protease Inhibitors of Urinary Extracellular Vesicles in Diabetic Nephropathy

**DOI:** 10.1155/2015/289734

**Published:** 2015-03-19

**Authors:** Luca Musante, Dorota Tataruch, Dongfeng Gu, Xinyu Liu, Carol Forsblom, Per-Henrik Groop, Harry Holthofer

**Affiliations:** ^1^Centre for Bioanalytical Sciences (CBAS), Dublin City University, Dublin 9, Ireland; ^2^Folkhälsan Institute of Genetics, Folkhälsan Research Center, 00100 Helsinki, Finland; ^3^Department of Medicine, Division of Nephrology, Helsinki University Central Hospital, 00100 Helsinki, Finland; ^4^Diabetes and Obesity, Research Program Unit, University of Helsinki, 00100 Helsinki, Finland; ^5^Baker IDI Heart and Diabetes Institute, Melbourne, VIC 3004, Australia

## Abstract

Diabetic nephropathy (DN) is one of the major complications of diabetes mellitus (DM), leads to chronic kidney disease (CKD), and, ultimately, is the main cause for end-stage kidney disease (ESKD). Beyond urinary albumin, no reliable biomarkers are available for accurate early diagnostics. Urinary extracellular vesicles (UEVs) have recently emerged as an interesting source of diagnostic and prognostic disease biomarkers. Here we used a protease and respective protease inhibitor array to profile urines of type 1 diabetes patients at different stages of kidney involvement. Urine samples were divided into groups based on the level of albuminuria and UEVs isolated by hydrostatic dialysis and screened for relative changes of 34 different proteases and 32 protease inhibitors, respectively. Interestingly, myeloblastin and its natural inhibitor elafin showed an increase in the normo- and microalbuminuric groups. Similarly, a characteristic pattern was observed in the array of protease inhibitors, with a marked increase of cystatin B, natural inhibitor of cathepsins L, H, and B as well as of neutrophil gelatinase-associated Lipocalin (NGAL) in the normoalbuminuric group. This study shows for the first time the distinctive alterations in comprehensive protease profiles of UEVs in diabetic nephropathy and uncovers intriguing mechanistic, prognostic, and diagnostic features of kidney damage in diabetes.

## 1. Introduction

Diabetes mellitus (DM) has emerged as one of the major global health problems and a heavy burden for all healthcare systems [1]. Current predictions estimate that by 2025 more than 480 million people globally will have an altered glucose tolerance and 380 million will have developed type 2 diabetes [2]. Among diabetic complications diabetic nephropathy (DN) has already become the leading cause of end-stage kidney disease (ESKD) worldwide [3, 4]. Moreover, progressive decline of kidney function is associated with an increase in all-cause mortality and severe cardiovascular complications in patients with diabetes [5–7]. Despite important advances in understanding, for example, the molecular pathways associated with the pathogenesis of DN [8, 9], the clinical management of patients, and pharmacological treatments to protect the kidney function are not completely satisfactory [10, 11].

The diagnosis of DN is based on clinical parameters including the measurement of urinary albumin excretion rate (AER), assessment of glomerular filtration rate (GFR) [12], and registering end-organ complications (retinopathy or neuropathy). Leakage of albumin into urine (albuminuria) has been the golden marker to indirectly indicate the integrity of the glomerular filtration barrier and as an index of kidney functionality. Depending on severity of damage, the level of albumin found in urine increases in a linear fashion. Patients are commonly stratified as* normoalbuminuric* (<20 *μ*g per minute or <30 mg/24 hours),* microalbuminuric* (20–200 *μ*g per minute or 30–300 mg/24 hours), or* macroalbuminuric* (>200 *μ*g per minute or >300 mg/24 hours) [13, 14]. In the diabetic patient, the onset of microalbuminuria proceeding to macroalbuminuria typically appears between 5 to 15 years and 15 to 25 years from the onset of diabetes [15, 16]. However, in patients with type 1 diabetes the decline of GFR is not always in concordance with the level of albuminuria and GFR reduces without clear signs of albuminuria and conversely reversion from microalbuminuria to normoalbuminuria can happen [17]. These evidences suggest that more than 1 pathway may well be involved in the development of DN and surrogate biomarkers in support of albuminuria are badly necessary to predict the progression of DN.

In the last decade urinary extracellular vesicles (UEVs) have gained considerable research interest due to their content of potential key molecules for intercellular communication and the possible use as source of biomarkers [18–20]. More in general cells secrete a surprising variety of vesicles such as exosomes, microvesicles, exosome-like vesicles, apoptotic blebs, and retrovirus-like particles (RLPs) into the extracellular space [21, 22] apparently reflecting intracellular processes. Thus, they provide a lucrative approach to better define the molecular events associated with metabolic disturbances as in diabetes. Moreover, UEVs have already shown to provide a promising source of biomarkers and their full potential has still to be utilised [23–25]. In this study we reveal, for the first time, a comprehensive analysis of proteases and proteases inhibitors in UEVs isolated from healthy subjects and DN in type 1 diabetes.

## 2. Methods

### 2.1. Urine Samples

Control urine samples were collected from twelve healthy volunteers among the laboratory staff, aged 20–40, in accordance with ethical protocols of the Dublin City University. First morning void urine was processed within 3 h without addition of protease inhibitors. Urine was anonymously tested by Combur 10 Test D dipstick (Roche Diagnostics, Basel, Switzerland) for the following: specific gravity, leucocytes, nitrites, proteins, glucose, ketones, urobilinogen, bilirubin, blood, and haemoglobin.

All patients participated in the Finnish Diabetic Nephropathy (FinnDiane) study, a nationwide multicenter study with the aim of identifying genetic and clinical risk factors for diabetic nephropathy in type 1 diabetes. The study protocol is in accordance with the Declaration of Helsinki, and it has been approved by the local ethics committee in each participating study centre. Urinary albumin excretion rate (AER) was determined in 24 h urine collections by immunoturbidimetry (Pharmacia, Uppsala, Sweden). The renal status was defined based on AER in at least two of three collections. Patients were divided by AER categorically.

### 2.2. Vesicle Purification

UEVs were purified by a hydrostatic filtration dialysis (*HFD*) system recently developed in our group [26]. Briefly, control and DN urine samples were centrifuged at a relative centrifugal force (RCF) of 2,000 g calculated at maximum radius of 160 mm in a swing bucket rotor Benchtop Universal 320 centrifuge (Hettich Zentrifugen, Tuttlingen, Germany) for 30 min at room temperature (RT) (without using break).

The supernatant (SN) was poured in a funnel connected to dialysis membrane with a molecular weight cutoff (MWCO) of 1,000 kDa as shown in supplemental Figure 1(S) (see Supplementary Material available online at http://dx.doi.org/10.1155/2015/289734). The hydrostatic pressure of the analyte solution is strong enough to push the solvent through the mesh of dialysis membrane carrying all the analytes below the MWCO (*HFDb*). Thus, after the first step of concentration (~3 mL) the funnel is refilled with 200 mL of deionised filtrate (0.22 *μ*m) water to rinse what is left in the concentrated urine until the volume of ~5 mL was reached again. This concentration process is called “hydrostatic filtration dialysis.” The retained solution above the 1,000 kDa cutoff (*HFDa*) was used for all the following experiments.

### 2.3. Protein Assay, Gel Electrophoresis, and Western Blot

Protein quantification was determined by Coomassie [27] microassay. Proteins were separated by SDS-PAGE employing freshly cast T 6–18%, C 2.6% gradient resolving gel (80 mm × 50 mm × 1.5 mm) [28]. Protein amounts (based on the Bradford assay) were first dried by vacuum concentration and then resuspended in a strong chaotropic solution made of the following: 7 M urea, 2 M thiourea, 5% (w/v) SDS, 40 mM Tris-HCl, pH 6.8, 0.5 mM ethylenediaminetetraacetic acid (EDTA), 20% (v/v) glycerol, and 50 mM dithiothreitol (DTT) in a ratio of 0.25 *μ*g of protein per *μ*L of solution [29]. Protein denaturation was obtained after an overnight (ON) incubation at RT. After electrophoresis the gels were either stained with colloidal Coomassie [30] or transferred to 0.45 *μ*m nitrocellulose membrane (Whatman, Springfield, UK) [31]. For Western blotting, membranes were saturated with Odyssey blocking buffer (LI-COR Biosciences, Lincoln, MA) and incubated with specific antibody as follows: rabbit anti-tumour suppressor gene (*TSG101*) (Sigma Aldrich, Dorset, UK), rabbit anti-ubiquitin (Dako, Glostrup, Denmark), and mouse anti-proteinase 3 (R&D Biosystem, Minneapolis, MN). After 6 washes in PBS-Tween (0.1%, v/v) membranes were incubated with infrared dye-coupled secondary antibody with a fluorescent tag (an emission of either 680 nm or 700 nm) (LI-COR Biosciences) dilution 1 : 5000 2 h incubation RT. Determination of molecular weight of all bands of interest and quantification of the signal were performed by Odyssey Infrared Laser Scanner software (LI-COR Biosciences).

### 2.4. Proteases and Protease Inhibitor Profiles

Proteome profiler human protease array kit and proteome profiler human protease inhibitor array kit (R&D Systems) were used to detect 34 proteases and 32 inhibitors, respectively. HFDa pooled fractions were dried by vacuum concentration (miVac speed trap, GeneVac Ltd, Ipswich, UK) and resuspended in 50 *μ*L of 0.3% (w/v) sodium dodecyl sulphate (SDS) for 2 hours. Samples were diluted to 1 mL with Array buffer 6 plus 0.5 mL of Array buffer 4 (both buffers from R&D Systems). Fifteen microliters of reconstituted detection antibody cocktail was added to the sample solution and incubated for 1 hour at RT in end-over-end agitation. Mix sample-antibody solutions were incubated overnight at 6°C with the membrane on a rocking platform shaker. After three 10-minute washes with 1x buffer, the membranes were incubated with infrared dye-coupled streptavidin with a fluorescent tag emitting at 800 nm (LI-COR Biosciences), 1 : 2000 dilution for 1 h at room temperature on the rocking platform shaker. After three × 10 min wash with 1x Wash Buffer the membrane slides were acquired by Odyssey Infrared Laser Scanner (LI-COR Biosciences) and relative quantification analysis was performed by Odyssey Infrared Laser Scanner software (LI-COR Biosciences).

### 2.5. Gelatin Zymography

Gelatin zymography was performed using gradient sodium dodecyl sulfate gel electrophoresis (SDS-PAGE, T = 6–18% C = 2.6%) copolymerized with 2 mg/mL pig skin gelatin type B (Sigma). UEVs (5 *μ*g per group) were incubated overnight in nonreducing Laemmli buffer [28]. After electrophoresis, gels were washed twice for 30 minutes in 2.5% (v/v) Triton X-100 at 4°C and were incubated overnight in collagenase buffer (50 mM Tris–HCl, pH 7.8 with 5 mM CaCl_2_ at 37°C). Gels were stained with Coomassie BlueR 250 (Bio-Rad) in 40% (v/v) methanol and 10% (v/v) acetic acid for 1 hour and were destained in the same solution without dye. Gelatinase activities were visualized as distinct bands, indicating proteolysis of the substrate [32].

### 2.6. Spectrophotometric Assay for Protease Activities

The proteolytic activities of specific group of proteases in normo-, micro- and macroalbuminuric HFDa fractions were assayed using 0.1 mM glycyl-prolyl-p-nitroanilide (GP-pNA) and 0.25 mM N-benzoyl-proline-phenylalanine-arginine-p-nitroanilide (Be-PFR-pNA), N-methoxysuccinyl-alanine-alanine-proline-valine-p-nitroanilide (Me-AAPV-pNA), and succinyl-alanine-alanine-proline-phenylalanine p-nitroanilide (Suc-AAPFpNA) chromogenic substrate of chromogenic substrates to establish activity of DPP IV, Kallikreins, proteinase 3, and cathepsins, respectively [23, 33–35]. Assays were performed for 20 min under same conditions of 100 mM Tris-HCl buffer pH 7.8 with addition of 5 mM CaCl_2_ except for GP-pNA, for which 100 mM Tris-HCl buffer pH 8.6 was used. Incubations were performed at 37°C for 20 min in 96-well flat bottom microplates (Greiner Bio One Kremsmüster, Austria). Reactions were stopped by 30% (v/v) final concentration of acetic acid. Results were read using 410 nm wavelength in Biotek PowerWave XS device (BioTek Instruments Inc., Winooski, VT). The specific activity has been defined as *μ*mole of 4-nitroaniline released per minute by one milligram of the protease under the assay conditions. Assays were performed for intact vesicles (without treatment) and after delipidation [36]. Briefly, an equivalent of 100 *μ*g of proteins from each sample was resuspended in milliQ water in total volume of 375 *μ*L. Next, 675 *μ*L of delipidation solution made of 60% (v/v) diisopropyl ether (DIPE) and 40% (v/v) butanol were added. Samples were vortexed and centrifuged for 10 min at 5000 rpm at RT. Upper phase was removed; 1125 *μ*L of DIPE was added to aqueous phase and mixed end-over-end for 5 min at RT. Samples were then centrifuged at 5000 rpm for 5 min in RT and the upper phase was discarded while tubes were placed under a fume hood to remove traces of an organic phase. Bradford assay was performed to establish protein concentration and samples were diluted to obtain an optimal concentration for colorimetric assay.

## 3. Results

**Figure 1:**
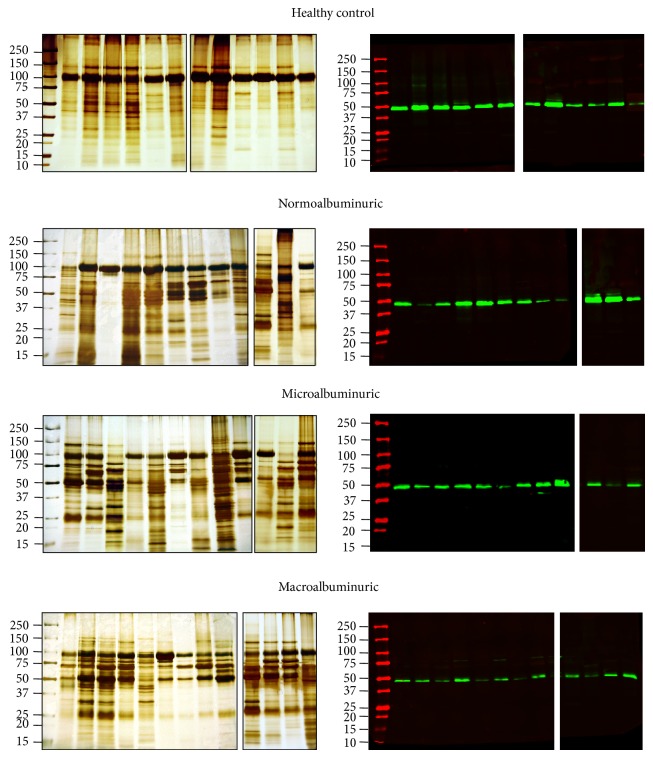
SDS-PAGE protein pattern and* TSG101* detection: Immunodetection of the exosome marker protein* TSG101* from the same order in the silver staining gels. Four *μ*g of protein (Bradford assay) was loaded per sample in each lane. Molecular weights are expressed in kilo Dalton.

In the pilot study 12 (6 males and 6 females) control volunteers provided 15 mL of midstream first morning urine. Thirty seven urine samples from diabetes patients, with different levels of albumin-proteinuria were studied. After enriching UEVs by hydrostatic filtration dialysis, samples were assayed in electrophoresis and Western blots for general protein patterns and detection of the respective vesicle markers including tumor suppressor gene 101 (*TSG101*) specific for exosome vesicles (Figure 1).

### 3.1. UEVs Characterization

**Figure 2:**
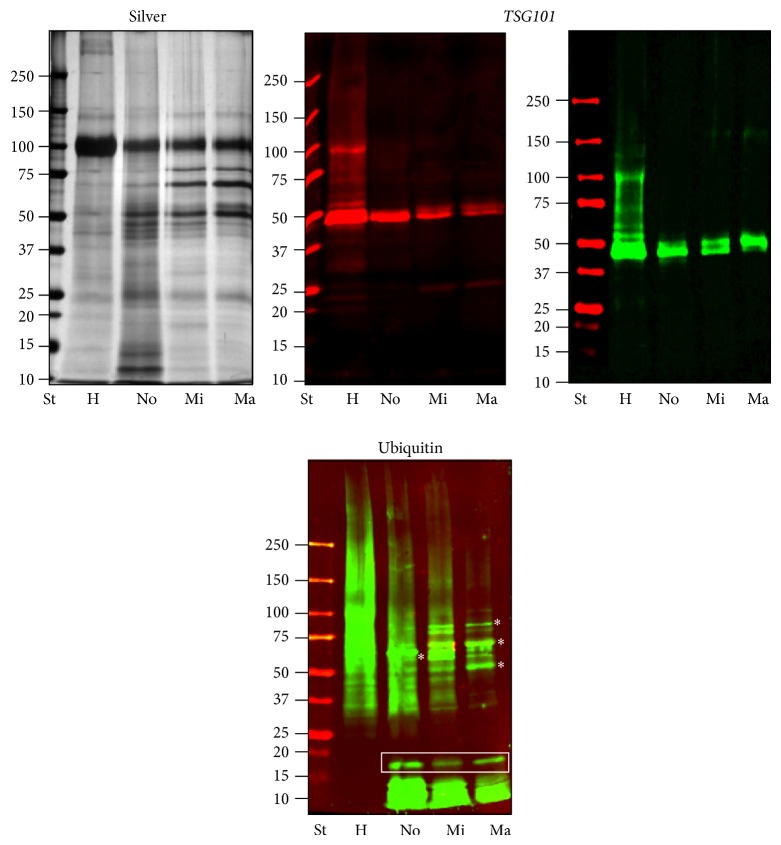
SDS-PAGE protein pattern,* TSG101*, and ubiquitin detection of pooled samples. Two *μ*g of protein (Bradford assay) was loaded per sample in each lane. Molecular weights are expressed in kilo Dalton. H: healthy control, N: normoalbuminuric, Mi: microalbuminuric, and Ma: macroalbuminuric.

Silver stained gels showed a good overlapping pattern with a moderate interindividual variability, most likely due to variable amounts of Tamm-Horsfall Protein (THP). Interestingly,* TSG101* assay also showed a progressive signal decrease in the micro- and macroalbuminuric groups, respectively. This trend was even more evident when urine pools were created for the protease and protease inhibitor arrays (Figure 2). Moreover, a detectable shift of the apparent* TSG101* molecular weight (MW) was observed when pools were run in adjacent lanes in the same gel. This most likely reflects changes in posttranslational modifications of exosome components during disease course. These results were confirmed in two independent Western blots, the second of which was carried out with an optimised gradient gel to have a better separation in the* TSG101* molecular weight (MW) region. In order to investigate this alteration in detail we assayed the ubiquitination state of exosomes. Although the precise molecular mechanism of the vesicle formation and protein recruitment needs to be fully elucidated, it seems apparent that this posttranslation modification (PTM) faithfully reflects the disease pathogenesis. Interestingly, ubiquitination is involved in a variety of cellular processes, including protein sorting and translocation inside the vesicle lumen during vesicle biogenesis as well as in protein degradation [37]. In support of this, Western blotting revealed a specific ubiquitination pattern in the DN groups. Anti-ubiquitin antibody used in this screening recognised free ubiquitin and monoubiquitinated protein. In all the DN groups it is possible to observe a strong signal at 8.5 kDa corresponding to the monomeric ubiquitin and 17 kDa (white rectangle) which is absent in the healthy control. Moreover a specific pattern in the normo-, micro-, and macroalbuminuric groups with an apparently relative changing for some bands (asterisk) is visible between 50 and 75 kDa.

### 3.2. Proteases Array and Relative Quantification

**Figure 3:**
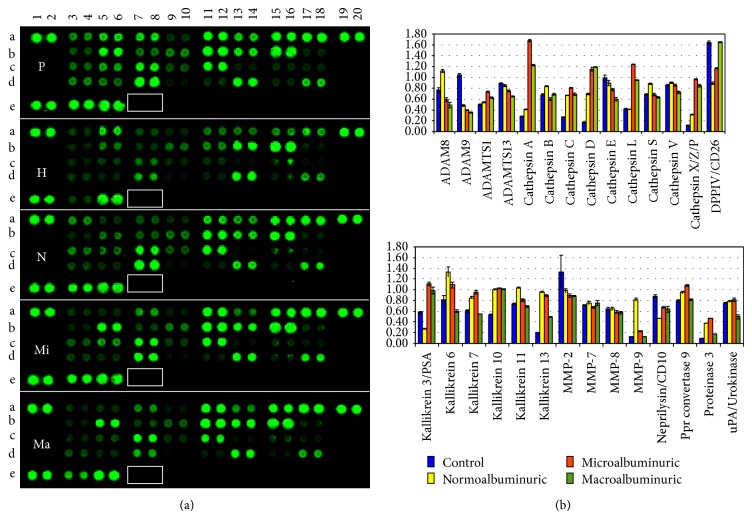
Protease array. (a) Nitrocellulose membrane slides were acquired at the same time as fluorescent intensity. (b) Relative quantification of positive protease (signal-to-noise ratio ≥3) was normalised by a pool made of all the samples included in the analysis. P: pool, H: healthy control, N: normoalbuminuric, Mi: microalbuminuric, and Ma: macroalbuminuric.

Despite the fact that the challenges of using sample pools have been thoroughly debated, pools for each study group were created to overcome substantial limitation such as a relative protein recovery from a limited volume of urine. Moreover a superpool made of the same amount of protein for each HFDa sample was created to normalise the relative quantitative changing of the proteases array (Figure 3(a)) and overcome the lack of a housekeeping protein.

Membrane slides were analyzed at the same time adjusting the intensity of the laser to reach the limit of saturation for the most abundant spot. The spots in positions A1,2, A19,20, and E1,2 represent the positive controls. The absence of spots in position E 7,8 (white rectangular), which is the negative control, indicates the specificity of the antibody cocktail.

Table 1 reports changes of ±1.5-fold of the proteases, with respect to the healthy control. The complete list is available in a spreadsheet as supplemental Table 1. A positive signal of 29 proteases was detected out of 34, out of which 16 showed a significant change during progression of DN.  Figure 3(b) shows the plots as bar chart of the fluorescent intensity (F.I.) for each protease normalised by the F.I. of the superpool. Positive spots were considered as those with a detection limit (signal-to-noise ratio) ≥3.

A set of proteases appear to progressively increase in different groups of DN patients (especially for the cathepsin family with a progressive increase of A, C, D, L, and Z) while only cathepsin E seems to decrease following a trend which becomes significant (less than 1.5-fold) in the macroalbuminuric group.

In the family of Kallikreins, Kallikrein 3/PSA, Kallikrein 10, Kallikrein 13, and Kallikrein 6 could be biased by the gender distribution of samples in the groups. Metalloprotease* MMP9* is abundantly present in the normoalbuminuric group while* MMP2* showed a progressive decrease reaching the threshold of −1.5-fold in the micro- and macroalbuminuric groups. Other proteases which showed an interesting trend are DDP IV targets of gliptins and proteinase 3 (*PRTN3*) or myeloblastin. DDP IV decreased in the normoalbuminic group and then increased during progression of DN while proteinase 3 (*PRTN3*) has an opposite trend with a marked increase in the normoalbuminuric and microalbuminuric group to reach a normal level in the macroalbuminuric group.

### 3.3. Protease Activities

**Figure 4:**
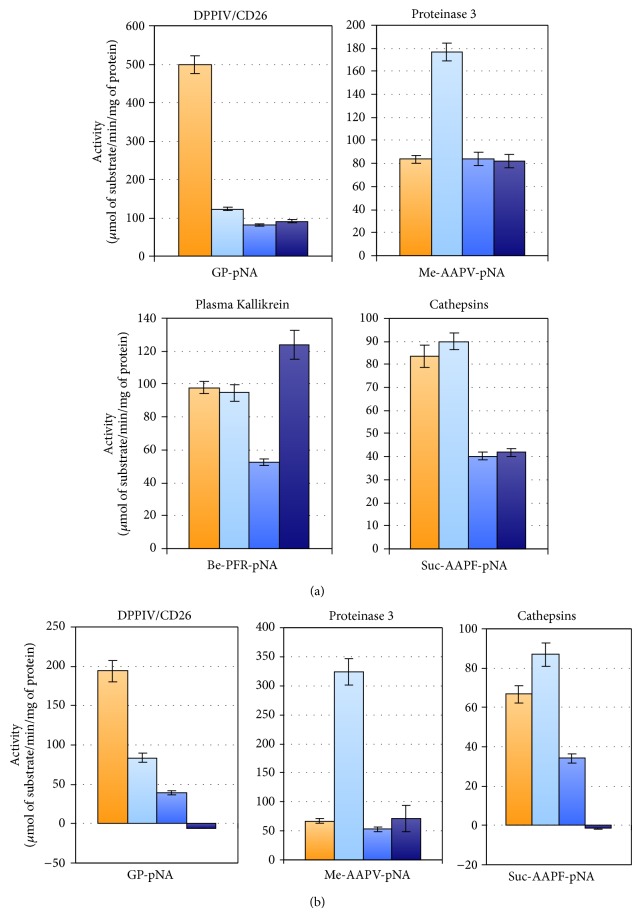
Protease chromogenic activity. Protease activities were assessed by specific chromogenic substrates for DDPIV Gly-Pro-p-nitroanilide (GP-pNA), leukocyte proteinase 3, and elastase (N-methoxysuccinyl Ala-Ala-Pro-Val-p-nitroanilide M-MeAAPV-pNA). Two *μ*g of protein (Bradford assay) was used per replica. The bar represents the standard deviation of the technical triplicate.

**Figure 5:**
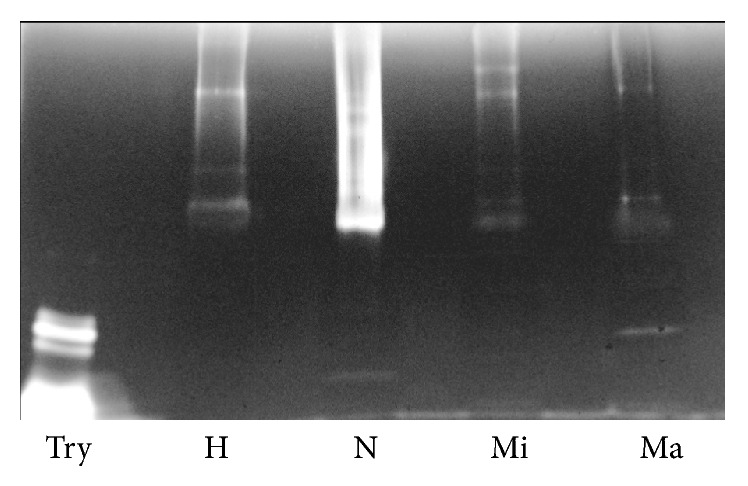
Zymography SDS-PAGE of vesicle-associated gelatinases. One *μ*g of protein per each pool (Bradford assay) was loaded in each lane. Gel was incubated at 37°C for 16 hours. Try: Trypsin (50 ng), H: healthy, N: normoalbuminuric, Mi: microalbuminuric, and Ma: macroalbuminuric pools.

Beside the relative quantitative amount we checked the activity of some of these proteases by gelatine zymography for metalloproteases (Figure 4) which confirmed a clear increase of gelatinase activity in the normoalbuminuric group and by chromogenic substrates for DPP IV, Kallikreins, cathepsins, and* PRTN3* (Figure 5). Spectrophotometric assays were performed in native condition and after organic delipidation to release proteases which can be localized in the vesicle lumen. After delipidation only the Kallikrein family was significantly affected (full loss of activity) while all the other protease activities maintained the same profile. Although there is a substantial increase of the cathepsin expression in the protease array, the colorimetric assay showed a decrease of the activity in the DN groups. Moreover, it is interesting to notice that the activity of DPP IV was much lower in the DN groups independently of the levels detected in the array. Proteinase 3 activity was high in the normoalbuminuric group but in the other groups it remained unchanged despite the higher levels especially in the microalbuminuric group. Since proteomic profiling of UEVs reported the presence of several protease inhibitors [18, 19], such discrepancy between activity and expression levels could be caused by the presence of protease inhibitors.

### 3.4. Protease Inhibitor Array and Relative Quantification

**Figure 6:**
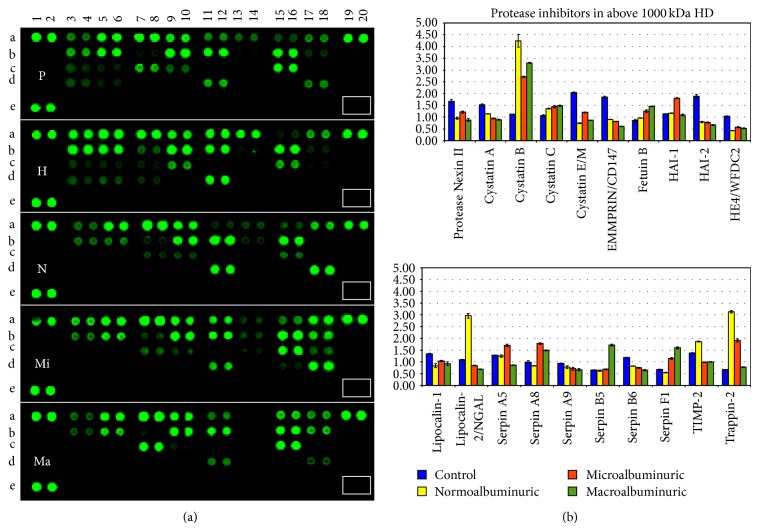
Protease inhibitors array. (a) Nitrocellulose membrane slides were acquired at the same time as fluorescent intensity. (b) Relative quantification of positive protease (signal-to-noise ratio ≥3) normalised by a pool made of all the samples included in the analysis. P: pool, H: healthy control, N: normoalbuminuric, M: microalbuminuric, and Ma: macroalbuminuric.

Based on the same assay, a protease inhibitor array to screen the expression of protease inhibitors was performed. Out of 32 inhibitors 19 were present in the same pool, and 15 showed more than 1.5-fold change (Table 2 and Figure 6). Also in this array we can identify different trends. Firstly, 6 inhibitors appeared to be more abundant (cystatin B, fetuin B, angiotensinogen, serpin A8, serpin F1, and elafin) progressively in the 3 diabetic groups. Those with decreased amounts showed either a progressive decrease which becomes significant in the micro- and/or macroalbuminuric groups (e.g., serpin B5 and TIMP-2) or very low amount in all the 3 DN groups (cystatin C E/M, EMMPRIN/CD147, HA-2, and HE4/WFDC2). Finally, two protease inhibitors (Lipocalin-2/NGAL and Protease Nexin II) showed a peculiar increase in the normoalbuminuric group and a sharp decrease in the macroalbuminuric cohort.

**Figure 7:**
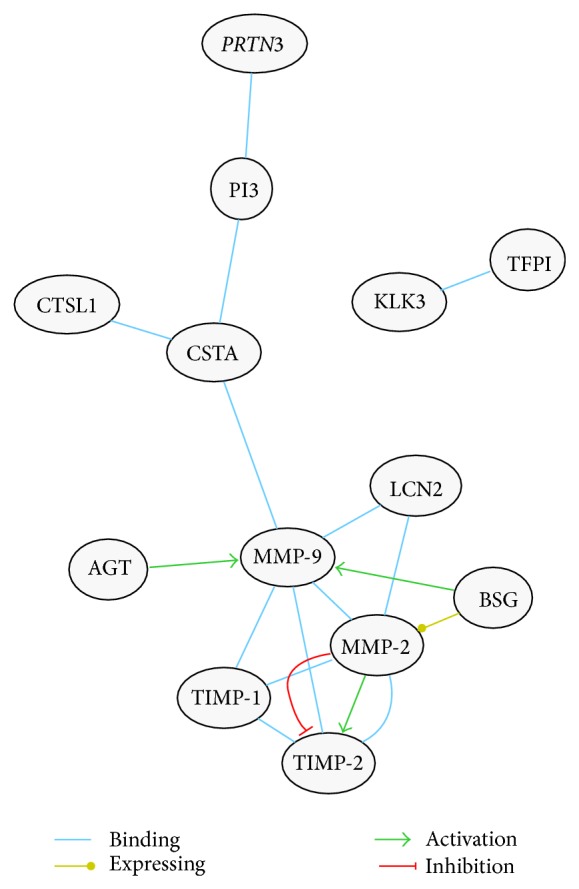
Urinary and kidney network map. A biological network of interaction using 30 proteases and proteases inhibitor entries extrapolated from the respective arrays was analysed by KUPKB [38]. Selected proteins were 1.5-fold up- or downregulated with respect to the healthy control. According to their intracellular pathways, several proteases can bind and degrade also endogenous inhibitors. This type of binding and interactions can add further complexity to the respective networks, and proteolytic signals themselves may end up in multiple directions. Accordingly, cathepsin L inactivates serpinA1 [75] and MMPs can inactivate a variety of serpins [76]. Conversely, some cystatins interact with MMP-9, still preserving the catalytic function after autodegradation [77]. Interestingly, angiotensinogen (AGT) decreases, through the action of AGT II, the transcription of MMP-1, MMP-2, TIMP-1, TIMP-2, and TIMP-3 but increases MMP-9 in human cardiomyocytes [78].

**Figure 8:**
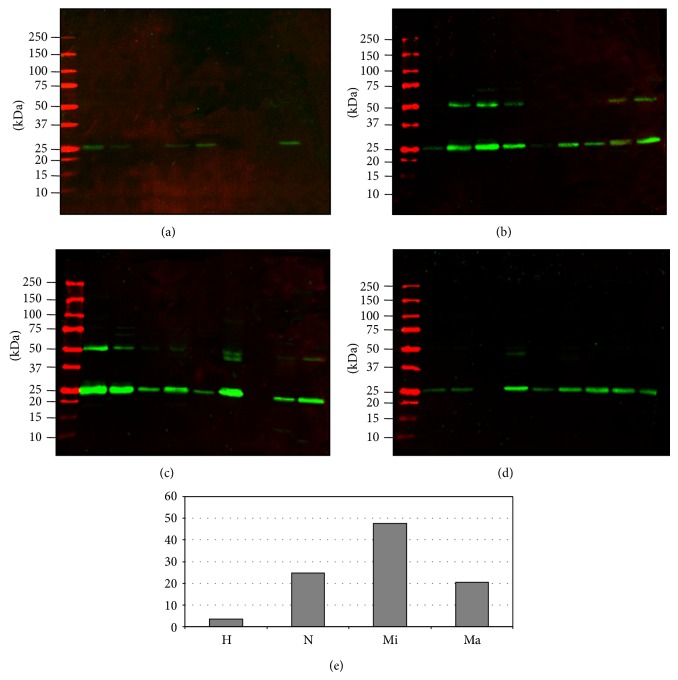
Myeloblastin (PRNT3) immunodetection. Two *μ*g of protein (Bradford assay) was loaded per sample in each lane. Nitrocellulose membranes were acquired at the same time as fluorescent intensity. (a) Healthy Control. (b) Normoalbuminuric. (c) Microalbuminuric. (d) Macroalbuminuric. (e) Average of the fluorescent intensity of each group. Molecular weights are expressed in kilo Dalton. H: healthy control, N: normoalbuminuric, Mi: microalbuminuric, and Ma: macroalbuminuric.

In order to establish whether there is any functional interconnection between proteases and protease inhibitors we used the Kidney & Urinary Pathway knowledge Base (KUPKB) [38] designed to collect data set from scientific publications and other datasets related specifically to renal diseases. The query of the KUPKB algorithm provided the protein network presented in Figure 7. Out of 30 entries which are up- or downregulated by 1.5-fold in the protease and protease inhibitor arrays, 11 entities were found to be connected as a network together. Within the network we have found that cathepsin L via cystatin A is connected to the metalloproteases 9 and 2 whose dysregulation is potentially involved in the progression of DN [39]. Interestingly, PRNT-3 mRNA was reported to be upregulated in type 2 diabetic nephropathy [40]. Thus, a further screening in Western blot was carried out evaluating the full cohorts of samples as shown in Figure 8 qualitatively confirming the array data.

## 4. Discussion

Diabetic nephropathy (DN) is a major complication of diabetic patients, constituting the leading cause of ESKD and considerably increasing cardiovascular risk and associated mortality. Defining the pathophysiologic mechanisms in DN is necessary to better understand the disease and identify new targets for therapeutic intervention. Kidney lesions can start to develop early during the quiescent period when the patient is still normoalbuminuric and with normal glomerular filtration rate (GFR) [17].

Microalbuminuria has a solid role in the clinical practice and management of diabetic kidney disease (DKD). However, an increasing number of studies have shown that decrease of GFR can occur independently from the presence of albuminuria or progression to macroalbuminuria and therefore the utility of albuminuria itself has been questioned [41]. Thus, in addition to albumin excretion rate (AER) and GFR, selected biomarkers for the detection of early functional abnormalities have been proposed and recently reviewed by Macisaac et al. [42].

Recently urinary exosomes or, more in general, urinary extracellular vesicles (UEVs) have been actively studied for their biological role as a potentially new cell-to-cell communication system. Despite the partly overlapping and confusing nomenclature, EVs can be divided into various main categories with respect to their secretory pathway [21, 22]. Thus, exosome vesicles are formed by inward invagination of the endosomal membrane in an ubiquitin-dependent mechanism which requires the Endosomal Sorting Complex Required for Transport (ESCRT) machinery. Exosomes are then released in the extracellular space when multivesicular bodies (MVBs) of late endosomes fuse with the plasma membrane and release their intraluminal vesicle cargo [43]. Microvesicles are shed vesicles or membrane particles which derive directly from the outward budding of plasma membrane in response to a variety of pathophysiological stimuli [44, 45]. Furthermore, EVs can mediate intercellular communication by delivering elements of genetic content from one cell to another in a paracellular fashion [46]. Thus, UEVs faithfully reflect the antigenic characteristics of their parent cells thus making them excellent cell-type specific. This has led to an explosion of interest in EVs as potential source of biomarkers as mirror of the pathophysiology of the cell of origin [19, 47]. Thus, UEVs can provide a good platform as a surrogate of a “fluid biopsy” to support the clinical management of, for example, diabetic patients, assuming the DN kidney damage is reflected in the repertoire of UEVs with a specific fingerprint at each stage of disease progression.

The first aim of this study was to isolate and characterize UEVs from healthy donors and 3 cohorts of DN grouped according to the AER using a new isolation method developed in our group [26]. This is based on a hybrid ultrafiltration-dialysis system which enriches vesicles without the interference of soluble protein like human serum albumin (HSA). As shown in Figure 1 for all the samples under investigation, the main protein present in the UEVs is Tamm-Horsfall Protein (THP). THP is abundant in the healthy controls and it progressively decreases in the DN groups. No evidence of massive presence of human serum albumin (HSA) even in the macroalbuminuric group was seen. As expected, more prominent bands in the microalbuminuric and macroalbuminuric groups are visible at 62, 52, and 25 kDa and these correspond to the heavy and light chains of immunoglobulin (Ig) isotypes *α*, *γ*, *κ*, and/or *λ* chains.

Immunoglobulins have been identified in the proteome profiling of UEVs [18, 19] and elevated concentration of IgG and IgA in urine has been proposed as a novel mechanism of kidney damage independent from charge and size impairment in early DN [48, 49]. More dedicated studies are necessary to investigate the role of vesicles-Ig interaction in DN for their role.

Detection of the exosomal marker* TSG101* confirmed the abundance of UEVs in the HFDa fractions. Interestingly, we noticed a progressive decrease of the signal during worsening of AER. Additionally, a shift of the molecular weight of* TSG101* was observed (Figure 2).* TSG101* can be acetylated [50] and/or ubiquitylated [51] and as component of the ESCRT-I machinery it binds ubiquitylated protein to sort it out into vesicles during its biogenesis to form the multivesicular body (MVB) which then either can release its vesicle cargo (exosomes) outside the cell and/or fuse with lysosome for degradation [52]. Thus, the ubiquitinated pattern of UEVs in the healthy and DN groups was checked by Western blot utilising a polyclonal antibody which recognised ubiquitin and monoubiquitinated proteins (Figure 3). Interestingly, the DN samples, at different stages of disease, provided a well defined and characteristic ubiquitome. This result along with decrease of* TSG101* signal suggests that an impairment of the endosome/vesicle trafficking happens in the early stages of disease. Ubiquitin, more in general the ubiquitin-proteasome, plays a pivotal role in the degradation of misfolded proteins, concentration, and turnover of cellular proteins. Thus the ubiquitin-proteasome system works in synergy with lysosomal proteases, caspases, calpains, and separases to generate shorter polypeptides accessible to the proteasomes system for a full protein degradation [53]. Our results show convincingly that there are major intracellular changes reacting to altered microenvironment and this is reflected in the void UEVs.

Proteases cover an important role not only in the pathogenesis of DN [39] but more in general as the dynamic reaction obviously needed to keep the intracellular homeostasis, in our case to fight back the hyperglycaemia induced changes [54, 55]. In this study we wanted to focus our attention on proteases first and their well established inhibitors and how they are represented in the UEVs as indicators of intracellular events.

Sixteen and nineteen proteases and inhibitors, respectively, changed by ±1.5-fold with respect to the healthy group. Cathepsins of the A, C, D, L, and XZP classes were found as markedly more abundant in the DN groups. The only one less represented is cathepsin E, while cathepsins B, S, and V did not show any change. Cathepsins are lysosomal proteases, which can exert their proteolytic activity in the extracellular space in extracellular matrix (ECM) remodeling, and in synergy with MMP are implicated in the development of renal diseases (CRDs) [56, 57]. Beside the increased level of cathepsin L the protease inhibitors array showed a concomitant increase of cystatin B a thiol proteinase inhibitor which, reversibly, binds cathepsin L [58]. Interestingly, neutrophil gelatinase-associated Lipocalin (NGAL) binds and preserves metalloproteinase 9 (MMP-9) from degradation thus supporting its gelatinase activity [59]. Increase of urinary excretion of NGAL-MMP-9 has been described in diabetic subjects in a gender-specific manner [60]. In our study proteases, protease inhibitors arrays, and their functional verification with zymography showed that this association is also reflected inside the UEV fraction. Intriguingly, whether these findings show a “spillover” reflection of the cellular reaction to elevated glucose content or a mechanism to allow specific targeting to more downstream sites of protease needs should be studied in detail.

Increase of the urinary level of DPP IV associated with microvesicles is correlated with the worsening of DN [23]. In our array, we found that the level of DPP IV decreases in the normoalbuminuric group and then starts to progressively increase in the samples from patients with micro- and macroalbuminuria. Independently of the levels DPP IV, its functional activity is markedly lower in the 3 DN groups than in control.

Sun and colleagues [23] reported an increased excretion of DPP IV associated with microvesicles in type 2 diabetic patients. Their results are in contrast with the result of our screening but the methodological approach can explain such a difference. While in our study we collected the whole amount of UEVs retained in the dialysis tube, they enriched DPP IV using a specific monoclonal antibody anti-AD-1 (leucine aminopeptidase). The authors reported it to be expressed on the brush border of the proximal tubular in the cortex (S1, S2 segments) and in the outer medulla and in the medullary rays (S3 segments). However, DPP IV is widely expressed also in the glomeruli [61] and an increase of DPP IV activity in podocytes is correlated with kidney injury [62, 63]. Of course additional studies are necessary to better evaluate changes in the DPP IV levels in microvesicles.

In order to analyze the link between the proteases and their inhibitor we used the Kidney & Urinary Pathway knowledge Base (KUPKB) [38] to specifically focus on proteins/genes relative to pathways in the kidneys. Notably, a distinct protein network as shown in Figure 7 was observed. In addition to the MMPs and their relative inhibitors, two relatively new proteins emerged which are potentially involved in the complexity of DN pathogenesis: myeloblastin and its natural inhibitor Trappin-2. Myeloblastin or proteinase 3 (*PRTN3*) is a serine protease present in granules and the cell surface of neutrophils and monocytes. It can function in a membrane bound or soluble form. It has pleiotropic effects and multiple substrates including extracellular matrix and cytokines [64, 65] and it can be found in urine [66]. Recruitment of neutrophils in glomeruli and the release of* PRTN3* potentially lead to endothelial dysfunction [67]. More recently podocytes have shown to actively regulate neutrophil recruitment through action of glomerular endothelial cells [68].* PRTN3* binds to the endothelial surface but it can be also internalised inducing apoptosis [69, 70]. Moreover,* PRTN3* has been proposed as an inflammatory enzyme able to digest insulin-like growth factor 1 (*IGF-1*) and the insulin-like growth factor-binding protein-3 (*IGFBP3*) and promote glomerular inflammation in type 2 diabetes. Interestingly, endothelial dysfunction and inflammation have been recently proposed as predictors of DN in type 1 and 2 diabetes [71, 72]. Our results showed that also these mechanisms can be monitored by detecting a combination of selected proteases and their inhibitors. The protease array (Figure 3), verified with the functional chromogenic activity (Figure 5) and screening patients (Figure 8), confirmed an increase of the level and activity already in the normoalbuminuric group: in patients with changes induced by hyperglycemia but with no signs of permanent kidney damage yet. Indirectly we can generalize that hyperglycemia leads also to inflammation, contributes to the complexity of changes, and can be the ultimate functional component for apoptosis and autophagy [73]. This is supported by the dysregulated ubiquitination mirrored in the UEVs (Figure 2). This is interesting as very recently it has been reported that the ubiquitination-dependent of coactivator-associated arginine methyltransferase 1 (*CARM1*) can mediate podocyte apoptosis in DN [74]. A more comprehensive analysis of the distinct proteases and their association to their preferential pathophysiological processes is warranted.

In conclusion, UEVs have a strong potential as key elements for a fluid kidney biopsy by reflecting the respective pathophysiology, exact mechanisms involved, and often also information on the exact domain of the nephron affected. Consequently, they also carry a great potential to develop distinct inhibitors/activators. However, more detailed studies and, most preferably, correlating findings with those from tissue biopsies and clinical parameters are imperative. Here we show that the new hydrostatic filtration dialysis method offers an unbiased approach for vesicle enrichment from urine and their subsequent use for distinct purposes of protease inhibitor studies.

## Supplementary Material

Supplemental Figure 1: Hydrostatic filtration dialysis system, assembling and function. Pictures show how to assemble the system with basic laboratory tools and the main phases of filtration to enrich urinary vesicles step by step.
Supplemental Table 1: Full list of proteases and protease inhibitors arrays. All proteases and inhibitors with a noise to signal ratio ≤ 3 are in red. Proteases and protease inhibitors with ± 1.5 fold changes with respect to the healthy control group are highlighted in yellow and green respectively.

## Figures and Tables

**Table 1:** Proteases array coordinate, features, and changing fold.

Coordinates	Protein name	Uniprot number	Gene name	Vesiclepedia	Isoform specificity	Normo	Micro	Macro
A5, A6	ADAM9	Q13443	ADAM9	*√*	Ectodomain	−2.16	−2.62	−2.92
A11, A12	Cathepsin A	P10619	CTSA	*√*	Proform and active	1.46	5.93	4.34
A15, A16	Cathepsin C	P53634	CTSC	*√*	Proform and active	2.47	2.98	2.50
A17, A18	Cathepsin D	P07339	CTSD	*√*	Proform and active	4.00	6.62	6.88
B3, B4	Cathepsin E	P14091	CTSE	*√* ^*^	Proform and active	−1.11	−1.29	−1.63
B5, B6	Cathepsin L	P07711	CTSL1	*√*	Proform and active	−1.01	2.95	2.27
B11, B12	Cathepsin X/Z/P	Q9UBR2	CTSZ	*√*	Proform and active	2.63	8.13	7.19
B13, B14	DPPIV/CD26	P27487	*DPP4 *	*√*	Ectodomain	−1.84	−1.41	1.00
B15, B16	Kallikrein 3/PSA	P07288	KLK3	*√*	Proform and active	−2.13	1.91	1.68
C3, C4	Kallikrein 6	Q92876	KLK6	*√*	Proform and active	1.63	1.34	−1.35
C7, C8	Kallikrein 10	O43240	KLK10	*√* ^‡^	Proform and active	1.87	1.93	1.90
C11, C12	Kallikrein 13	Q9UKR3	KLK13	X	Proform and active	4.70	4.38	2.44
C15, C16	MMP-2	P08253	MMP2	*√*	Proform and active	−1.36	−1.50	−1.52
D7, D8	MMP-9	P14780	MMP9	*√*	Proform and active	6.40	1.83	−1.02
D13, D14	Neprilysin/CD10	P08473	MME	*√*	Ectodomain	−1.89	−1.30	−1.37
E3, E4	Proteinase 3	P24158	*PRTN3 *	*√*	Active	4.15	5.20	1.96

^*^Reported in vesiclepedia database in mouse and rat species.

^‡^Reported in vesiclepedia database as mRNA.

**Table 2:** Protease inhibitor array, coordinates, features, and changing fold.

Coordinates	Protein name	Uniprot number	Gene name	Vesiclepedia	Normo	Micro	Macro
A3, A4	Protease Nexin II	P05067	APP	*√*	−1.75	−1.37	−1.90
A5, A6	Cystatin A	P01040	CSTA	*√*	−1.35	−1.62	−1.73
A7, A8	Cystatin B	P04080	CSTB	*√*	3.74	2.39	2.91
A11, A12	Cystatin E/M	Q15828	CST6	*√*	−2.75	−1.71	−2.36
A13, A14	EMMPRIN/CD147	P35613	BSG	*√*	−2.05	−2.27	−3.08
A15, A16	Fetuin B	Q9UGM5	FETUB	X	1.11	1.44	1.68
B3, B4	HAI-2	O43291	SPINT2	*√* ^‡^	−2.38	−2.43	−2.82
B5, B6	HE4/WFDC2	Q14508	WAP 5	*√* ^‡^	−2.44	−1.81	−1.99
B9, B10	Lipocalin-1	P31025	LCN1	*√*	−1.62	−1.30	−1.47
B11, B12	Lipocalin-2/NGAL	P80188	LCN2	*√*	2.72	−1.30	−1.61
B17, B18	Serpin A8	P01019	AGT	*√*	−1.19	1.79	1.50
C7, C8	Serpin B5	P36952	SERPINB5	*√*	−1.03	1.06	2.67
C9, C10	Serpin B6	P35237	SERPINB6	*√*	−1.43	−1.57	−1.81
C15, C16	Serpin F1	P36955	SERPINEF1	*√*	−1.23	1.69	2.36
D5, D6	TFPI	P10646	TFPI	*√*	−1.44	−1.46	−1.54
D7, D8	TFPI-2	P48307	TFPI2	X	−1.31	−1.44	−1.61
D9, D10	TIMP-1	P01033	TIMP1	*√*	−1.31	−1.14	−1.57
D11, D12	TIMP-2	P16035	TIMP2	*√*	1.29	−1.47	−1.86
D17, D18	Elafin	P19957	PI3	X	4.62	2.84	1.15

^‡^Reported in vesiclepedia database as mRNA.
