# Supplementary figures and images for: Proteases and Protease Inhibitors of Urinary Extracellular Vesicles in Diabetic Nephropathy
Source: J Diabetes Res. 2015 Mar 19;2015:289734. doi: 10.1155/2015/289734 (PMC4383158; doi:10.1155/2015/289734)

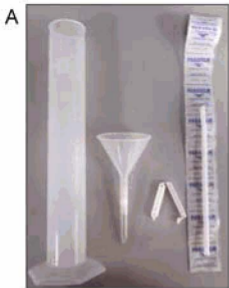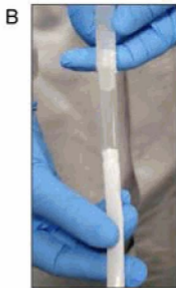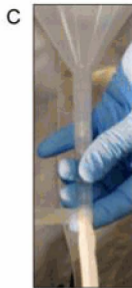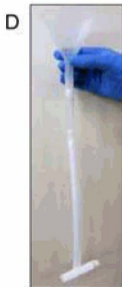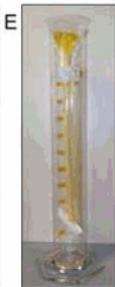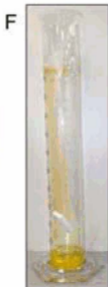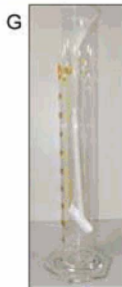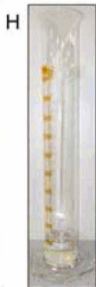

Supplement: Supplementary file 1 — Supplemental Figure 1: Hydrostatic filtration dialysis system, assembling and function. Pictures show how to assemble the system with basic laboratory tools and the main phases of filtration to enrich urinary vesicles step by step. Supplemental Table 1: Full list of proteases and protease inhibitors arrays. All proteases and inhibitors with a noise to signal ratio ≤ 3 are in red. Proteases and protease inhibitors with ± 1.5 fold changes with respect to the healthy control group are highlighted in yellow and green respectively. [file 289734.f1.pdf]
